# Targeting the GDF15 Signalling for Obesity Treatment: Recent Advances and Emerging Challenges

**DOI:** 10.1111/jcmm.70251

**Published:** 2024-12-19

**Authors:** Jincheng Zhang, Jingquan Sun, Jielang Li, Hongwei Xia

**Affiliations:** ^1^ Division of Abdominal Tumor Multimodality Treatment, Department of Medical Oncology, Cancer Center and National Clinical Research Center for Geriatrics and Laboratory of Molecular Targeted Therapy in Oncology, Frontiers Science Center for Disease‐Related Molecular Network, West China Hospital Sichuan University Chengdu China; ^2^ School of Physical Education and Sports Sichuan University Chengdu China; ^3^ Research Institute of Molecular Exercise Science Hungarian University of Sports Science Budapest Hungary

**Keywords:** appetite suppression, energy metabolism, GDF15, GFRAL, obesity, weight loss

## Abstract

The growth differentiation factor 15 (GDF15)–glial cell‐derived neurotrophic factor family receptor alpha‐like (GFRAL) pathway plays a crucial role in the regulation of metabolism, appetite and body weight control. Obesity is an increasingly prevalent chronic disease worldwide, necessitating effective treatment strategies. Recent preclinical and clinical studies have highlighted that targeting the GDF15‐GFRAL signalling pathway is a promising approach for treating obesity, particularly because it has minimal impact on skeletal muscle mass, which is essential to preserve during weight loss. Given its distinctive mechanisms, the GDF15‐GFRAL axis represents an attractive target for addressing various metabolic disorders, especially obesity. In this review, we will explore how the GDF15‐GFRAL axis is regulated, its distribution in the body and its role in the regulation of metabolism, appetite and obesity. Additionally, we will discuss recent advances and potential challenges in targeting the GDF15‐GFRAL axis for obesity treatment.

**Abbreviations**
AMPK: AMP‐activated protein kinase
AP: area postrema
ATF: activating transcription factors
ATGL: adipose triglyceride lipase
ATM: adipose tissue macrophages
BAT: brown adipose tissue
CHOP: C/EBP homologous protein
CRP: C‐reactive protein
EGR1: early growth response protein 1
eIF2α: eukaryotic initiation factor 2 alpha
FAO: fatty acid oxidation
FDA: Food and Drug Administration
GDF15: growth differentiation factor 15
GLCE: glucuronyl C5‐epimerase
GLP1: glucagon‐like peptide 1
GFRAL: GDNF family receptor alpha‐like
HSL: hormone‐sensitive lipase
HIF‐1α: hypoxia‐inducible factor‐1α
HFD: high‐fat diet
ISR: integrated stress response
KEAP1: Kelch‐1ike ECH‐associated Protein l
KLF4: Kluber‐like factor‐4
MT1‐MMP/MMP14: membrane‐bound matrix metalloproteinase 14
NTS: nucleus tractus solitarius
OXPHOS: oxidative phosphorylation
PERK: protein kinase R‐like (PKR‐like) endoplasmic reticulum kinase
PKA: protein kinase A
RET: rearranged during transfection
ROS: reactive oxygen species
TG: triglycerides
UPRmt: mitochondrial unfolded protein response
UCP1: uncoupling protein‐1
WAT: white adipose tissue

## 1 Introduction

Obesity is a highly prevalent chronic disease, affecting 650 million adults worldwide [1, 2]. Clinical complications of obesity affect many important organ systems and increase the risk of multiple metabolic‐related diseases [3], including cardiovascular disease [4], coronary heart disease [5], heart failure [6], diabetes mellitus and hypertension [7]. The current treatment strategies for obesity include broadly lifestyle interventions, surgery and medications [8]. Reducing excess body fat through single lifestyle interventions, such as exercise or diet restriction, might pose challenges due to prolonged compliance, adherence difficulties and a gradual onset of efficacy [9]. Clinical guidelines recommend lifestyle‐assisted drug therapy for patients with a body mass index (BMI) of 27 or greater. Metabolic surgery is indicated for patients with Grade II obesity (BMI ≥ 35 to < 40 kg/m^2^), Grade III obesity (BMI > 40 kg/m^2^) and Type 2 Diabetes (T2DM). Common bariatric procedures include Roux‐en‐Y gastric bypass and sleeve gastrectomy, but surgical treatments usually lead to vitamin deficiencies, surgical complications, gastrooesophageal reflux, dumping syndrome and high costs [10].

The use of existing drugs is restricted because of their modest efficacy, safety concerns and high costs [11]. The Endocrine Society guidelines suggest that the obesity patients with active cardiovascular disease should consider lorcaserin or orlistat as potential treatment options, as these medications are less likely to increase the risk of cardiovascular events [12]. Recent clinical advances have revolutionised how we will treat obesity in the near future. Notably, metreleptin (a leptin analogue), setmelanotide (a melanocortin‐4 (MC4) receptor agonist), phentermine/topiramate (a norepinephrine agonist/g This figure was created using BioRender (https://biorender.com/) amma‐aminobutyric acid agonist and glutamate antagonist) and naltrexone/bupropion (an opioid receptor antagonist and dopamine/norepinephrine reuptake inhibitor), as well as the (glucagon‐like peptide 1) GLP1 agonists liraglutide and semaglutide, have been approved for the treatment of obesity [13]. In the realm of obesity treatment, liraglutide has gained approval at higher dosages and demonstrated significant weight loss effects in clinical trials. Similar to liraglutide, semaglutide operates by increasing insulin secretion, reducing glucagon release, slowing digestion and promoting satiety, contributing to improved glycaemic control. In clinical trials, semaglutide has shown remarkable weight loss outcomes among individuals with obesity, positioning it as a promising avenue for obesity management [13]. Tirzepatide, a new generation GLP1 and glucose‐dependent insulinotropic polypeptide (GIP) dual agonist, exhibited a weight loss of about 20%, with about one‐third of patients achieving a weight loss of 25% or more, almost achieving surgical results and it recently received Food and Drug Administration (FDA) approval for the treatment of diabetes and obesity [14, 15, 16].

Although the GLP1 agonists, such as liraglutide, semaglutide and tirzepatide, have demonstrated clinical effectiveness in addressing obesity, it is important to note that these drugs have also been associated with certain side effects during dose escalation, including constipation, diarrhoea, nausea, dyspepsia and sarcopenia [13, 16]. Therefore, it is crucial for us to identify novel mechanisms involved in obesity and develop innovative therapeutic strategies that could effectively reduce weight without causing side effects.

The transforming growth factor‐β (TGF‐β) superfamily consists of an expanding group of growth and differentiation factors that are involved in many important biological processes, such as growth, differentiation, wound healing, metabolism and inflammation [17, 18]. Macrophage inhibitory cytokine‐1 (MIC1) is a divergent member of the TGF‐β superfamily [19] and it was also historically known as PLAB [20], NAG1 [21], PTGFB [22] and PDF [23]. Recent studies showed that the growth differentiation factor 15 (GDF15) played an important role in the regulation of metabolism, appetite, body weight control and anorexia–cachexia syndrome of cancer [18].

The binding of GDF15 to its receptor glial cell‐derived neurotrophic factor family receptor alpha‐like (GFRAL) has been reported to decrease food intake and thus lead to weight loss. These specific mechanisms make GDF15‐GFRAL pathway a potential target for treating many metabolic diseases, including obesity [24]. Several GDF15 analogues have shown promising efficiency in preclinical models, and some have entered clinical trials (NCT04199351, NCT04010786 and NCT03764774). In this review, we will summarise the molecular mechanisms of GDF15‐GFRAL pathway in obesity, and we will also discuss the efficacy of drugs that target the GDF15‐GFRAL pathway for the treatment of obesity and the potential challenges.

## 2 The Biology, Expression and Distribution of GDF15 and its Receptor

The *Gdf15* gene is located at the chromosomal locus 19p13.11 [25]. GDF15 is present in various forms, encompassing GDF15 premonomer, GDF15 predimer, peptide pre–N‐terminal fragment and mature dimer [26, 27]. The GDF15 protein is initially synthesised as a pre–pro‐GDF15 precursor, comprising 308 amino acids. This precursor protein subsequently undergoes cleavage by a furin‐like protease with an RXXR sequence to yield pro‐GDF15 (~30 kDa). Then the β‐arrestin 1 facilitates the translocation of pre‐GDF15 to the Golgi for cleavage and maturation, where it undergoes further cleavage by matrix metalloproteinase 26 (MMP26), paired alkaline amino acid cleavage enzyme 4 (PACE4) and three members of the subtilin‐like proprotein convertase family (PCSK3, PCSK5 and PCSK6). This process results in the production of mature GDF15 (112 amino acids) of approximately 13 kDa, which then forms homodimers through disulphide bonds [18, 19, 25, 28]. Unprocessed GDF15 precursors exhibit a rapid secretion, while intracellular processing leads to the secretion of mature GDF15 at a considerably slower pace, potentially via an alternative secretory pathway. The COOH‐terminal 47 amino acids within the prepropeptide are responsible for the efficient secretion of GDF15 precursors. Therefore, the intracellular processing of GDF15 plays a crucial role in regulating the secretion rate of GDF15 and the storage of its pro‐GDF15 substrate, depending on the presence or absence of its propeptide [29].

In the past years, the concept of GDF15‐induced reductions in appetite and body weight has been well established, although the exact mechanism remains elusive [30]. It was not until 2017 that several groups successfully characterised the GFRAL as the receptor for GDF15 nearly at the same time [31, 32, 33, 34]. GFRAL, a distal homologue of the GDNF family, plays a significant role in neuroprotection and brain development [35]. Quantitative PCR (QPCR) analysis showed that GFRAL was not expressed in mouse peripheral tissues and was only expressed in the area postrema (AP) of the hindbrain. In human tissues, GFRAL mRNA was detected in the brain, testis and adipose tissue. Immunohistochemistry (IHC) assays showed that the neurons with GFRAL‐positive expression were detected in the AP and nucleus of the solitary tract (NTS) [31]. However, the specific role of GFRAL in fat and testis tissues needs to be further elucidated.

**FIGURE 1:**
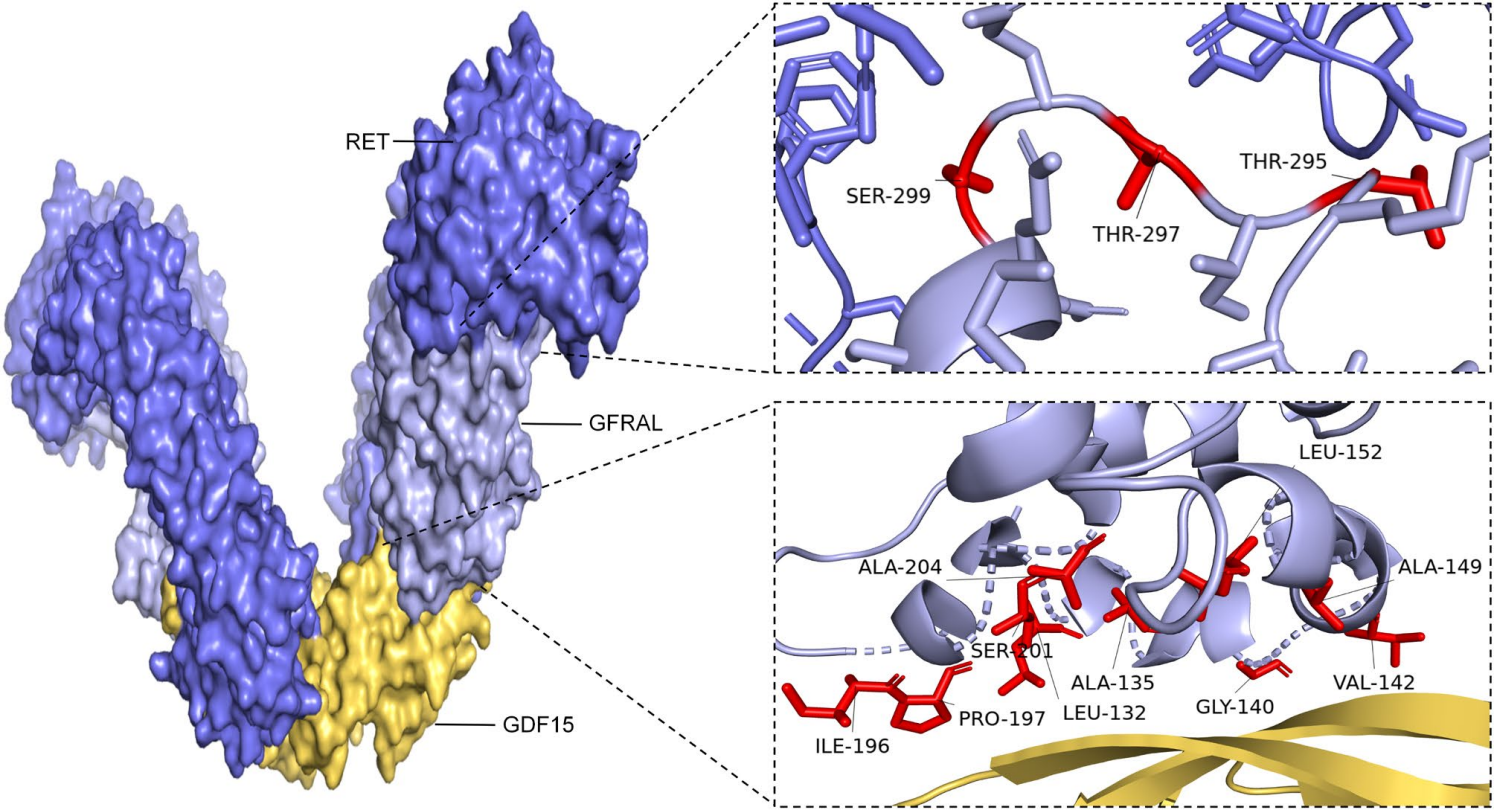
The 3D structural model depicting the interaction sites of GDF15 with its receptors GFRAL and RET. The interaction sites of GDF15 with GFRAL encompass specific amino acids, including Leu132, Ala135, Glu136, Val139, Gly140, Val142, Asn145, Ala149, Leu152, Lys153, Ile196, Pro197, Gln200, Ser201 and Ala204. Notably, Glu136, Asn145 and Gln200 play vital roles as residues engaged in hydrogen bonding with GDF15.However, despite these potential interaction sites being identified, current research lacks conclusive evidence to fully elucidate the specific functional roles of these residues in the GDF15‐GFRAL interaction. A therapeutic antagonist monoclonal antibody, 3P10, targeting GFRAL inhibits RET signalling by obstructing the GDF15‐driven interaction between RET and the cell surface GFRAL. Molecules associated with muscle atrophy exhibited alterations in the muscle tissue of homozygous mice treated with 3P10. Five key interacting residues in the GFRAL D3 structural domain (Trg294, Thr295, Thr297, Gln298 and Ser299) are shared between RET and 3P10. These sites may be implicated in skeletal muscle atrophy. The 3D structural model was created with PyMOL, utilising structure coordinates sourced from the Protein Data Bank (PDB IDs: 5VZ4 & 6Q2J).

Activation of brainstem neurons in the AP and NTS has been noted after intraperitoneal GDF15 injection in the male C57BL/6 mice. Importantly, the GDF15‐induced satiety effects were completely abolished upon excision of solely these two regions [36]. Structural analyses have illuminated that GFRAL is composed of 394‐amino‐acid residues, with a molecular weight of 44,518 Da [25]. GDF15 interacts with its receptors, GFRAL and rearranged during transfection (RET), at the sites as indicated by Jawed Akhtar Siddiqui et al. [25] and Rowena Suriben et al. [37] (Figure 1). The extracellular region of GFRAL is composed of three cysteine structural domains; the D2 structural domain contains a hydrophobic pocket that interacts with specific amino acid residues located in the finger‐like protrusions of GDF15 [33]. The transmembrane tyrosine kinase coreceptor RET is expressed in various tissues, except for the liver, kidney and adrenal gland [32]. The receptor complex comprising GFRAL and RET is distinct and does not overlap with other receptors. GDF15 induces the activation of neurons coexpressing GFRAL and RET in vivo, initiating a signalling cascade involving phosphorylated ribosomal protein S6 (pS6) [33], AKT, ERK and PLCγ in GFRAL‐expressing neurons. This cascade leads to reduced food intake, weight loss, improved glucose homeostasis [31] and induces nausea and vomiting [38]. In recent years, increasing evidence showed that the peripheral GDF15 could still execute its distinct function in various cells and organs that do not express the receptor GFRAL. It has been reported that the lipolytic effects of GDF15 precursors are seemingly reliant on unidentified receptors [39]. So further studies are needed to screen and identify novel GDF15 receptors and investigate its functions in obesity.

GDF15 is widely expressed across various organs and tissues, such as placenta, liver, skeletal muscle, adipose tissue, myocardium, choroid plexus epithelium of the adult brain, intestinal mucosa, bronchus, bronchi, submandibular secretory ducts and lactating mammary gland [40, 41, 42, 43]. In comparison, GDF15 mRNA expression showed lower levels in the pancreas, prostate and foetal lung [44]. The expression of GDF15 is regulated by several factors, such as inflammation, infection, tumours, ionising radiation and various drugs [45]. Yet, the precise role of GDF15 in distinct tissues or organs under normal physiological circumstances remains an important subject that warrants further exploration [45].

**FIGURE 2:**
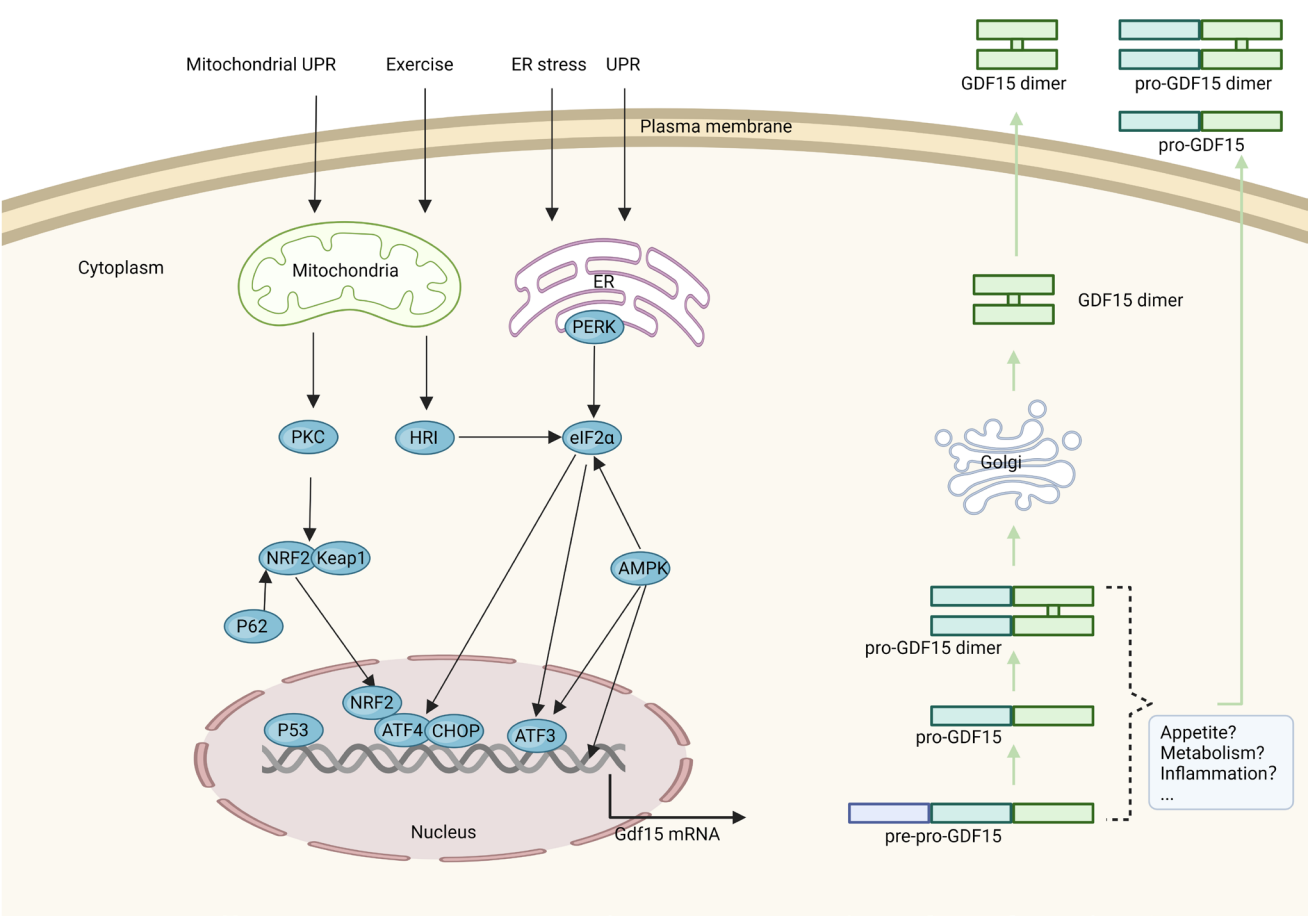
Diagram of the mechanisms of GDF15 expression, maturation and secretion. Various stress stimuli, such as mitochondrial unfolded protein response (UPRmt), exercise, and endoplasmic reticulum (ER) stress, activate the integrated stress response (ISR) pathway to induce the expression of GDF15. Regulators such as heme‐regulated inhibitor (HRI), protein kinase R (PKR), PKR‐like ER kinase (PERK), and general control non‐depressible 2 (GCN2) phosphorylate eIF2α, activating ATF4 and CHOP, which form heterodimers to upregulate GDF15 expression [52, 53]. It has been reported that metformin [54] and AMP‐activated protein kinase (AMPK) agonists (AICAR, R419, A769662) induce GDF15 expression through activation of the AMPK signaling pathway and the PERK–eIF2α–ATF3/4–CHOP axis [55]. Berberine enhances GDF15 expression via the PKC, ERK, and GSK3β pathways and induces ATF3 expression through P53 [56]. In brown adipose tissue (BAT), berberine activates pERK, initiating eIF2α phosphorylation, which subsequently increases ATF4, CHOP, and FGF21 expression [43]. Colchicine activates P62, leading to KEAP1 (Kelch‐like ECH‐associated protein 1) degradation and subsequent NRF2 activation, thereby stimulating GDF15 secretion [57]. Apigenin disrupts the binding of NRF2 to KEAP1, causing NRF2 degradation and stimulating GDF15 secretion [58]. GDF15 is primarily synthesized in the cytoplasm as an inactive precursor protein (pre‐pro‐GDF15), which is subsequently cleaved to form pro‐GDF15. The pro‐GDF15 monomer forms pro‐GDF15 homodimers via disulfide bonds, which are then transported to the Golgi, where they are cleaved to form mature GDF15 dimers and secreted extracellularly. In certain cell types, GDF15 can also be secreted as pro‐GDF15 monomers or dimers. Dashed lines represent processes with unclear mechanisms [52]. Created in BioRender. Zhang, J. (2024) https://BioRender.com/u13e611.

In lean and healthy mice, the expression of Gdf15 mRNA was found to be relatively high in the kidney, liver, white adipose tissue (WAT), brown adipose tissue (BAT) and skeletal muscle [46], which is regulated by a diverse range of transcription factors, including glucuronyl C5‐epimerase (GLCE) [47], P53, Kluber‐like factor‐4 (KLF4), early growth response protein 1 (EGR1) [45], hypoxia‐inducible factor‐1α (HIF‐1α), activating transcription factors 3 and 4 (ATF3 and ATF4) and C/EBP homologous protein (CHOP) [48]. GDF15 secretion was observed in activated macrophages, while its expression was absent in quiescent macrophages [19]. It has been reported that GDF15 tends to accumulate in the liver before being released into the bloodstream [49] and possesses a circulating half‐life of 2–3 h [50, 51] (Figure 2).

Under physiological conditions, the levels of GDF15 in human and mouse blood are typically 0.1–1.2 ng/mL and 50–100 pg/mL, respectively [39, 52]. However, in obese mice, rats and humans, the circulating concentrations of GDF15 were significantly elevated. In female subjects, GDF15 serum levels were notably higher in the obese and T2DM groups compared to the control group and exhibited a positive correlation with body weight, body fat, serum triglycerides, glucose, haemoglobin Alc (HbAlc) and C‐reactive protein (CRP). Despite the notably increased levels of GDF15 in obese and diabetic patients, there was no observed weight loss [59]. Recent studies showed that the pharmacological GDF15, acting through its receptor GFRAL, suppresses appetite and inhibits voluntary running activity [60]. However, the physiological induction of GDF15 through exercise does not have the same effects, highlighting an inconsistency between the impacts of endogenous and exogenous GDF15 on the human body [60]. This phenomenon may be attributed to the adaptive response of elevated GDF15 in disease state, which lacks a clear physiological function [61]. It is possible that individuals with obesity and insulin resistance may present with compromised mitochondrial function [62]. The upregulation of GDF15 in obesity appears to serve as a compensatory mechanism for impaired muscle mitochondrial function [63]. The study indicates that GDF15 concentrations are elevated in obese mice, rats and humans [50]. The elevated expression of GDF15 did not yield harmful consequences across a range of organs, including the brain, heart, lung, liver, gallbladder, spleen, pancreas, kidney, skeletal muscle, stomach, duodenum, jejunum, ileum, colon, prostate, prepuce gland, skin and adipose tissue [50]. An alternative hypothesis posits that the membrane‐bound matrix metalloproteinase 14 (MT1‐MMP/MMP14) serves as an endogenous negative regulator of GFRAL, and that nutritional overload may promote MT1‐MMP to proteolytically inactivate GFRAL proteins, thereby impeding the GDF15‐GFRAL signalling pathway and ultimately diminishing the anorexigenic effects of GDF15 [39, 64]. Recently, a study has shown that in obesity, the accumulation of macrophages in adipose tissue triggers an elevation in GDF‐15 levels, a process that is exacerbated when coexisting with T2D [65]. Given our preliminary understanding of the biological function of GFRAL, it is possible that it may play an unanticipated role in the obese population [66]. GDF15 also exhibits a notable increase in a variety of pathological conditions, including cancer and cachexia [25]. Whether elevated plasma concentrations of GDF15 are beneficial or detrimental in obesity and other illnesses still needs further investigation.

## 3 The Central Role of GDF15‐GFRAL Pathway in Regulating Food Intake

**FIGURE 3:**
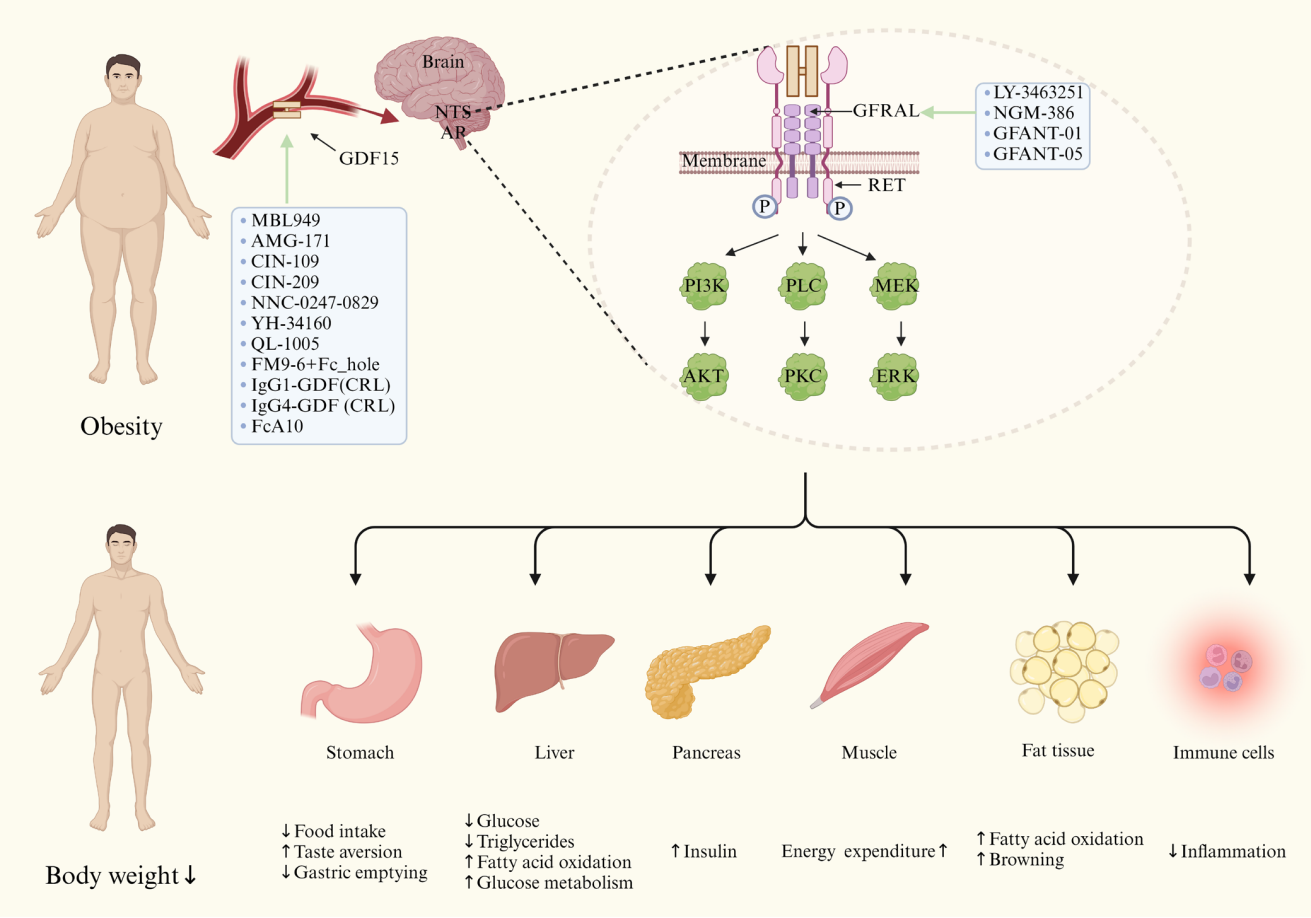
The effect of GDF15 analogues or GFRAL agonists on various tissues in obesity. The formation of the GDF15‐GFRAL‐RET signalling complex is essential for GDF15‐induced appetite suppression. Drugs targeting GDF15 include MBL949, AMG‐171, CIN‐109, CIN‐209, NNC0247‐0829, YH34160, QL1005, FM9‐6 + Fc_hole, IgG1‐GDF (CRL), IgG4‐GDF (CRL) and FcA10(−, L234A/L235A)‐(G4Q)4‐GDF15(N3Q/D5E). Those targeting GFRAL include LY3463251, NGM‐386, GFANT‐01 and GFANT‐05. Created in BioRender. Zhang, J. (2024) https://BioRender.com/b18k804.

The binding of GDF15 to its receptor GFRAL in the AP/NTS region of the hindbrain is essential for its metabolic effects on food intake and body weight in mice [34]. In the absence of GDF15 or inhibiting its activity, mice exhibited increased diet intake and obesity, as well as poorer blood glucose levels, insulin levels and glucose tolerance [67, 68, 69]. In contrast, obese rodents subjected to either transgenic or overexpression with *Gdf15* exhibited reductions in both food intake and body weight [31, 32, 50, 70, 71, 72, 73, 74, 75, 76, 77]. Similarly, treatment with recombinant GDF15 in nonhuman primates also reduced food intake and body weight [31, 50]. Additionally, the daily application of GDF15 to wild‐type mice led to decreased food consumption and subsequent loss of body weight. On the other hand, knockout (KO) of *Gfral* prevented the GDF15‐triggered declines in food intake, body weight and adiposity [32, 33] (Figure 3). Studies have shown that the activation of the GDF15‐GFRAL‐RET signalling pathway in the AP and NTS projects to the parabrachial nucleus (PBN), potentially inducing conditioned taste aversion (CTA), and to the arcuate nucleus (ARC) of the hypothalamus [52]. Additionally, other downstream targets in the paraventricular hypothalamic nucleus (PVH), central amygdala (CeA) and oval bed nucleus of the stria terminalis (ovBNST) are involved. This pathway modulates vagal and sympathetic nervous system activity, influencing food preferences, gastric emptying, hedonic hunger and the nausea and vomiting that affect food intake [61]. In addition to its role in the AP and NTS, GDF15 also activates a small population of neurons located in the caudal dorsal motor nucleus of the vagus (DMX). The primary function of DMX neurons is to transmit neural impulses to the gastrointestinal tract via the vagus nerve [36]. Furthermore, GDF15 predominantly activates noncatecholaminergic neurons and regulates the trafficking of the T‐type calcium channel α‐subunits CaV3.1 and CaV3.3, further promoting the release of the neurotransmitter glutamate in pyramidal neurons of the medial prefrontal cortex (mPFC). This mechanism plays a significant role in the regulation of appetite and energy metabolism [78].

The anorectic effect of GDF15 on GFRAL requires circulating levels of ≥ 400 pg/mL. Exogenous GDF15 only induces anorexic effects at high doses, while endogenous GDF15 needs to be elevated two to three times above baseline levels to generate anorexic effects [39]. The impact of GDF15 is influenced by its concentration, which is a crucial factor. In normal mice, low levels of GDF15 (< 200 pg/mL) were inadequate to induce food aversion and suppress appetite [79]. The GDF15‐GFRAL pathway does not appear to be associated with appetite regulation under normal physiological circumstances, as there are no significant changes in body weight, food intake and preference in mice lacking GDF15 and GFRAL [52]. Several studies have indicated that upregulation of GDF15 can reduce appetite and promote weight loss. However, in obese individuals, there is a notable increase in serum GDF15 expression without affecting appetite. Therefore, the effects of artificially introduced and disease‐associated endogenous elevation of GDF15 are inconsistent and these phenomena require further investigation.

## 4 GDF15 Sustains Energy Expenditure via GFRAL

Substantial evidence suggests that targeting the GDF15‐GFRAL pathway as a therapeutic approach might aid in maintaining skeletal muscle energy expenditure during periods of caloric restriction. Overexpression of GDF15 has been shown to suppress appetite, resulting in reduced energy intake [29, 33, 39, 80, 81, 82]. Conversely, downregulation of GDF15 is implicated in promoting the development of obesity [67, 68, 69, 72]. However, wild‐type mice exhibit lower energy intake after subcutaneous injection of GDF15, while the effect of GDF15 is not evident in mice lacking GFRAL. This suggests that GFRAL is necessary for GDF15‐driven anorexia and weight loss [31, 39].

Notably, GDF15 has been identified as a vital mediator for mild mitochondrial uncoupling, leading to enhanced metabolic status [63]. When exposed to a high‐fat diet (HFD), the mice overexpressing the human Nag‐1/Gdf15 gene (hNag‐1 mice) exhibited notably elevated levels of oxygen consumption (VO_2_), oxygen utilisation and carbon dioxide production (VCO_2_) compared to their wild‐type counterparts. Interestingly, these changes occurred while maintaining consistent respiratory exchange ratios (RERs) during both daytime and nighttime cycles. Furthermore, hNag1 mice exhibited augmented heat production during both cycles. These observations indicate that hNag‐1 mice possess enhanced metabolic activity and energy expenditure, as substantiated by their increased oxygen utilisation and heat production [75]. Not surprisingly, the Gdf15 KO mice demonstrate reduced metabolic rates, as indicated by their decreased levels of oxygen content and calorie production in comparison to the wild‐type mice in the diet‐induced obesity (DIO) model [67].

A recent intriguing experiment has elucidated a novel mechanism by which the GDF15 signalling pathway stimulates energy expenditure. Administering GDF15 to wild‐type mice and mice lacking β1, β2 and β3 adrenergic receptors (β‐less mice) revealed that the β‐less mice exhibit resistance to GDF15‐induced reduction in RER and increase in fatty acid oxidation (FAO). These findings suggest that GDF15 enhances energy expenditure through the GFRAL–β‐adrenergic axis, thereby promoting weight loss. This axis augments FAO and futile calcium cycling in skeletal muscles, consequently elevating energy expenditure in skeletal muscles [51].

## 5 The Role of GDF15 in Mitochondria

Mitochondria plays a vital role in the regulation of cellular energy homeostasis. When mitochondria in BAT become impaired due to protein misfolding or dysfunction in oxidative phosphorylation (OXPHOS) complexes, the mitochondrial unfolded protein response (UPRmt) is activated [83]. This response partially overlaps with the integrated stress response (ISR) pathway [38]. Activation of UPRmt not only enhances protein stability within the mitochondria but also induces the production and secretion of GDF15. The GDF15 signal activates GFRAL neurons in the brain as well as sympathetic preganglionic neurons in the spinal cord, which in turn stimulates the browning of WAT and increases metabolic heat production. This compensatory mechanism allows WAT to help maintain energy balance through thermogenesis when BAT is compromised [83]. The hNag1 mice exhibited an upregulation in the expression of thermogenic genes (UCP1, PGC1α, ECH1, Cox8b, Dio2, Cyc1, PGC1β, PPARα and Elvol3) within the BAT. These alterations in gene expression correlate with an enhanced energy metabolism [75].

In C2C12 myotube cells, chemically induced UPRmt leads to a significant increase in GDF15 expression. Overexpression of ATF4, ATF5 and ATF6 does not notably alter the activity of the Gdf15 promoter. Notably, overexpression of CHOP results in a significant upregulation of both Gdf15 mRNA levels and promoter activity. Furthermore, phosphorylation of CHOP by P38 MAPK has been observed, indicating a potential role of the P38‐CHOP pathway in regulating GDF15 expression in response to mitochondrial stress [63]. The interplay of nuclear and mitochondrial interactions also relies on transcription factors such as NRF1, NRF2, PPARα and the PGC1 family of coactivators (including PGC1α, PGC1β and PRC). These interactions play a crucial role in energy metabolism, oxidative stress response and mitochondrial biogenesis [84]. ATP serves as the universal energy currency in all living cells, primarily synthesised within mitochondria through OXPHOS. The terminal and rate‐limiting step of the respiratory chain is catalysed by cytochrome c oxidase (COX), which functions as a regulatory hub within the OXPHOS process [85]. The administration of recombinant GDF15 led to a dose‐dependent elevation in basal respiration, ATP corespiration and maximal respiration rate in adult myocytes and hepatocytes. Furthermore, pretreatment with rGDF15 resulted in an augmentation of FAO in adult myocytes and hepatocytes [63].

## 6 The Role of GDF15 in Glucose Metabolism

KO of the GDF15 in DIO mice results in compromised blood glucose levels, insulin levels and plasma glucose tolerance [67]. Conversely, reintroducing the Gdf15 gene under an HFD has been shown to significantly enhance glucose tolerance and improve insulin responsiveness [72]. Multiple experiments have demonstrated that therapeutic administration of recombinant GDF15 protein could reduce body weight in obese rodents and primates, along with improvements in glucose tolerance [31, 32, 33, 34, 50, 67, 72]. GDF15 may contribute, in part, to the improvement of insulin resistance in HFD‐fed mice by potentially elevating serum adiponectin levels [72]. Furthermore, it has been observed that mice with *Gfral* gene KO exhibit reduced glucose tolerance and elevated insulin concentrations [31]. Recent studies have revealed that recombinant GDF15 enhances insulin secretion through the upregulation of the insulin secretion pathway in β cells in a GFRAL‐independent manner. A 6‐week regimen of high‐intensity exercise training has been shown to elevate circulating GDF15 levels, which correlates positively with the improvement of β‐cell function in individuals with T2DM [86]. However, further research is needed to elucidate how GDF15 influences other metabolic indicators and what role it plays in the pathogenesis of obesity.

## 7 The Role of GDF15 in Lipolysis

The GDF15 protein has been demonstrated to alleviate inflammation, promote thermogenesis and lipolysis, sustain AMPK activity as well as enhance insulin sensitivity and mitigate hepatic steatosis [39]. The recombinant GDF15 treatment could reduce the total WAT content and increase UCP‐1 mRNA in the inguinal WAT and BAT in the mice fed with HFD [72]. The study revealed a notable increase in GDF15 expression within various muscle tissues, including the tibialis anterior, gastrocnemius, quadriceps femoris, piriformis, diaphragm and oesophageal muscles in HSA‐Ucp1‐TG mice [87]. Upregulation of fat breakdown genes β3‐adrenoceptor (Adrb3), adipose triglyceride lipase (Atgl), and hormone‐sensitive lipase (Hsl) was detected in the WAT and BAT of Gdf15 transgenic mice. These changes in gene expression align with enhanced energy metabolism [75].

GDF15‐GFRAL signalling could influence sympathetic nervous system activity, resulting in increased expression of HSL and ATGL, heightened fat breakdown and reduced fat mass, ultimately leading to weight loss [43]. Excitingly, in vitro experiments using conditioned media demonstrate that GDF15 secreted by skeletal muscle specifically targets the GFRAL/RET receptors on human adipose tissue, thereby activating genes involved in fat breakdown metabolism [88]. In future studies, a more definitive understanding of the independent roles of GDF15‐GFRAL/RET in adipose tissues is needed. It has been suggested that the peripheral effects of GDF15 are mediated by the secretion of other forms of GDF15 that are different from mature dimers, such as monomeric pro‐GDF15 or dimeric proGDF15 [89]. Future studies are needed to reveal the effects of different forms of GDF15 on adipose tissue.

## 8 The Role of GDF15 in Inflammation

Inflammation plays a vital role in the pathological process of obesity, which was primarily developed from metabolic disorders in the adipose tissues. Adipocytes and infiltrating inflammatory cells, specifically adipose tissue macrophages (ATM) attracted by chemokines, release proinflammatory cytokines. Administration of recombinant GDF15 could notably reduce the mRNA expression of macrophage markers F4/80 and CD68a in HFD‐fed mice. Conversely, suppressing GDF15 in HFD‐fed mice resulted in increased infiltration of ATM and heightened inflammatory reactions [72]. The specific caspase‐1 inhibitor pralnacasan was found to reduce weight gain and improve insulin sensitivity in obese ob/ob mice [90]. Furthermore, the adipose tissue of Gdf15‐TG mice exhibited a decrease in the expression of IL18, IL1β and TNFα, accompanied by a significant reduction in serum leptin and insulin levels. Additionally, the expression of macrophage infiltration markers, namely, F4/80, CD11b and CD11c, was significantly diminished [73]. GDF15 reduces NAFLD and hepatic inflammation through a GFRAL‐dependent mechanism which is independent of reductions in food intake [51]. However, it has been suggested that the mature GDF15 dimer structure may not have anti‐inflammatory properties. Alternative forms of GDF15 may mediate the development of inflammation [80]. In the future, additional research is necessary to investigate the impact of various forms of GDF15 on cellular inflammation development in obesity.

## 9 Relationship Between GDF15 and GLP1

GLP1, primarily secreted from the intestinal cells in response to food intake, plays an important role in the regulation of appetite and glucolipid metabolism. It binds to a specific GLP1 receptor (GLP1R) which is expressed in several tissues, such as the pancreas, brain, kidney, lung, heart and major blood vessels. The primary target cell of GLP1 is the insulin‐secreting β‐cells, which play a crucial role in increasing insulin secretion in response to glucose. GLP1 activation helps to regulate blood glucose levels, slows down the emptying of the stomach and induces a feeling of fullness. This activation triggers the production of cAMP through the activation of adenylyl cyclase, leading to the activation of protein kinase A (PKA) and EPAC pathways. In the past decade, GLP1R agonists have been successfully used for managing T2DM in clinic [91]. GLP1R agonists, such as liraglutide and semaglutide, originally developed for the treatment of T2DM, have demonstrated significant effectiveness in managing obesity based on their incretin effects and have been approved by FDA for the treatment of obesity [92]. However, certain side effects, including constipation, diarrhoea, nausea, pancreatitis and dyspepsia, limit the efficacy [13].

GDF15 has been shown to reverse bulimia and obesity in obese rat models with MC4 receptor deficiency and leptin receptor deficiency. Additionally, GLP1 can activate neurons in the PBN, which involves brain stem cells expressing the GLP1R [33]. However, within the same study, it was also reported that the weight‐reducing effects of GDF15 are not contingent on GLP1, as weight loss induced by GDF15 could still be observed in mice lacking the Glp1r gene. On the other hand, weight loss driven by GLP1 remained intact in mice lacking the *Gfral* gene [33]. GDF15 level remained unchanged following treatment with liraglutide or lorcaserin in obesity patients, indicating that it does not directly participate in the metabolic feedback pathways activated by GLP1R agonists [93]. Loss of GDF15 or GFRAL signalling did not affect the ability of the GLP1R agonist liraglutide to reduce food intake. Similarly, loss of GLP1R signalling did not reduce the anorexic effect of GDF15. Interestingly, when mice were simultaneously treated with GDF15 agonist and liraglutide, a significant synergistic effect on weight loss was observed [94]. Notably, GDF15‐treated mice retained muscle mass while losing fat compared to the GLP1 agonist group. This distinction is of paramount significance as rapid muscle loss constitutes a well‐known side effect of common weight loss pharmaceuticals. Thus, a potential optimal approach could involve combining GDF15 with a GLP1R agonist or utilising unmodified GDF15 as a standalone therapy in humans [51].

However, another study presented conflicting findings, demonstrating that increased GDF15 expression in muscle actually resulted in a decrease in local muscle mass [95]. Furthermore, no correlation was observed between GDF15 levels and indicators of bone health, indicating that the impact of GDF15 on bone may be mediated through other pathways involving different tissues [96]. The implications of GDF15 treatment on skeletal muscle is still somewhat controversial and warrant further investigation. It is also crucial for future studies to explore strategies that prioritise the preservation of skeletal muscle mass during weight loss endeavours.

## 10 Therapeutic Implications of Endogenous and Exogenous GDF15

The endogenous GDF15 is naturally produced by the body under both physiological and pathological conditions and rises significantly in response to metabolic stress, such as intense exercise, fasting, acute HFD or thermoneutral conditions at 30°C [60] and disease states (e.g., cancer [25], obesity and Type 2 diabetes [97]). During exercise, GDF15 acts as a circulating myokine, increasing in response to muscle mitochondrial stress and dysfunction, which aids in muscle recovery and structural reorganisation. Following mitochondrial stress, GDF15 expression is elevated in skeletal muscle, supporting whole‐body metabolic homeostasis, improving insulin resistance and preventing DIO through the promotion of lipolysis and oxidative metabolism in the liver, muscle and WAT [98]. In human studies, simulated exercise raises GDF15 levels in muscle cells, which then promotes lipolysis through the GFRAL‐RET signalling pathway in human adipose tissue [88]. In contrast, animal studies show that forced running elevates endogenous GDF15 in both wild‐type and GFRAL KO mice, they exhibit similar baseline physiological traits, endurance, spontaneous activity, postexercise GDF15 levels and clearance rates [60]. Despite this apparent difference, it is noteworthy that GFRAL is not expressed in mouse adipose tissue, whereas its expression is detectable in human adipose tissue [88]. This suggests that the mechanism of action for exercise‐induced GDF15 may operate through peripheral receptors rather than GFRAL signalling in the brain. These findings further indicate that endogenous GDF15 may target a broad range of pathways. In early stages of obesity, GDF15, produced by macrophages in adipose tissue, can reduce weight gain, although this antiobesity effect appears to diminish as obesity progresses [65]. In various pathological states, such as cancer, cardiovascular diseases and chronic kidney disease, upregulation of endogenous GDF15 is considered an adaptive stress response, with potential anti‐inflammatory and protective functions [99]. However, studies also suggest that GDF15 contributes to ER stress‐induced β‐cell apoptosis, indicating that GDF15 inhibition may represent a novel strategy to promote β‐cell survival and support diabetes treatment [100]. Furthermore, endogenous GDF15 is widely utilised as a biomarker for the assessment of cardiovascular disease, diabetes, cancer, cognitive impairment, cachexia, ageing, mitochondrial dysfunction and lung diseases [101, 102].

Exogenous GDF15 refers to GDF15 introduced into the body through drugs or other interventions. Unlike endogenous GDF15, the therapeutic potential of exogenous GDF15 in obesity and metabolic diseases has been demonstrated by numerous animal and preclinical studies [103, 104, 105].

## 11 Pharmacological Interventions and Clinical Advances in Targeting the GDF15‐GFRAL Pathway for Obesity Treatment

### 11.1 MBL949

MBL949 is a half‐life–extended recombinant human GDF15 dimer. Animal studies have shown effective weight reduction primarily by reducing fat. In trials with overweight or obese adults, higher doses have demonstrated weight loss effects. In the Phase II trial, however, weight reduction was minimal after 14 weeks of intervention. Within the tested dosage range, MBL949 has been found to be safe for humans (NCT05199090) [105].

### 11.2 LY3463251

LY3463251, a GDF15 analogue, could act as a potent agonist at the GFRAL/RET receptor with prolonged pharmacokinetics. This drug was designed by Eli Lilly and Company and is a synthetic peptide that serves as a ligand for GDF15 fused with IgG4‐Fc. The Fc portion extends the drug's half‐life in circulation. Following a single subcutaneous injection, the substance exhibits half‐lives of 81 h in mice, 87 h in rats and 117 h in monkeys. Further investigations indicated that LY3463251 demonstrated the ability to decrease food intake and body weight in rodents and overweight nonprimates. LY3463251 injections showed a safety and pharmacokinetic profile in healthy individuals. Nevertheless, varying doses led to instances of nausea and vomiting among healthy participants. In the case of overweight and obese patients, LY3463251 yielded noteworthy declines in both food consumption and appetite associated with modest body weight loss independent of nausea and emesis (NCT03764774). Activation of the GFRAL/RET system worked to regulate energy equilibrium in humans. However, the reduction in body weight was surprisingly modest, potentially attributed to a dosage that may have been insufficient. The utilisation of the GDF15 system for clinical weight loss interventions still faces certain challenges and needs further investigation [103].

### 11.3 AMG‐171

AMG‐171, a GDF15 Fc fusion protein created by Amgen Inc., has undergone clinical testing. The preclinical studies revealed that administration of AMG‐171 led to decreased food intake and weight loss in mice, rats and obese cynomolgus monkeys. Additionally, this treatment was associated with delayed gastric emptying, changes in food preferences and enhancements in metabolic well‐being for the animals [50, 106]. Together, AMG‐171 has shown promising efficacy as an antiobesity agent in diverse animal models, and this drug has currently entered Phase I clinical trial. We are greatly looking forward to the future clinical results of this drug (NCT04199351) [50, 106].

### 11.4 CIN‐109/JNJ‐9090

The GDF15 analogue CIN‐109/JNJ‐9090, developed by CinFina (Janssen Sciences Ireland Unlimited Company), exhibited favourable tolerance in a Phase I clinical trial focusing on single ascending doses. Notably, this trial also highlighted a reduction in food intake. To advance the development of CIN‐109, following dose selection, the company plans to launch a Phase II study targeting overweight and obese adults within the United States [107]. Further clinical results of this drug are highly anticipated.

### 11.5 NNC0247‐0829

NNC0247‐0829, a novel GDF15 ligand modulator [108], is an investigational drug aimed at weight management in overweight or obese populations. This drug is currently undergoing Phase I clinical research (NCT04010786), and we are eagerly anticipating the forthcoming release of the research findings.

### 11.6 Compound H [CpdH]

CpdH is a GDF15 analogue designed to extend its half‐life, exhibiting linear and dose‐proportional pharmacokinetics with a terminal half‐life of approximately 8 days. Treatment with CpdH leads to a significant reduction in food intake and subsequent body weight. Moreover, CpdH demonstrates sustained effectiveness, as evidenced by its impact on first‐episode spontaneously obese nonhuman primates. Throughout a 12‐week span, CpdH consistently induced a dose‐dependent reduction in ad libitum dietary consumption, resulting in continuous weight loss. These findings suggest that CpdH holds promise as a potential candidate for effective clinical obesity pharmacotherapy [109].

### 11.7 YH34160

YH34160, developed by Yuhan Corporation, is an engineered variant of GDF15 fused with Fc, resulting in an extended half‐life and improved functional activity due to its heightened binding affinity to the GDF15 receptor GFRAL/RET. In the obesity mouse model, YH34160 exhibited a strong and lasting impact on reducing body weight. Interestingly, when YH34160 was combined with semaglutide, the weight loss effects were remarkably enhanced, resulting in more significant and substantial outcomes [110]. The latest clinical studies indicate that administration of liraglutide or naltrexone/bupropion does not affect GDF15 levels, suggesting that GDF15 mediates obesity treatment in a distinct way [111].

In addition to the above‐mentioned drugs, there are also several novel GDF15‐analogues currently being investigated, although the details about these drugs are limited. These antiobesity drugs include FM9‐6 + Fc_hole, IgG1‐GDF (CRL), IgG4‐GDF (CRL) and FcA10(−, L234A/L235A)‐(G4Q)4‐GDF15(N3Q/D5E). Further studies are needed to gather more information about their potential benefits and applications in animals and in clinic. Additionally, promising antiobesity medications, such as NGM‐386, GFANT‐01 and GFANT‐05, specifically focus on GFRAL. We are eagerly looking forward to the imminent release of further research findings on these innovative medications (https://pharma.bcpmdata.com/) (Table 1).

**TABLE 1:** Summary of the drugs targeting GDF15‐GFRAL pathway in obesity.

Drug name	Target	Condition	Phase	Clinical trial	R&D company
MBL949	GDF15	Obesity	Phase II	NCT05199090	Novartis Pharmaceuticals
AMG‐171	GDF15	Obesity	Phase I	NCT04199351	Amgen Inc
NNC‐0247‐0829 (LA‐GDF15 NN‐9215)	GDF15	Obesity	Phase I	NCT04010786	Novo Nordisk A/S
LY‐3463251	GDF15/GFRAL	Obesity	Phase I	NCT03764774	Eli Lilly & Co
CIN‐109 (JNJ‐9090)	GDF15	Obesity	Phase I	NA	Janssen Research & Development LLC
YH‐34160 (YHC‐2110)	GDF15	Obesity	Preclinical	NA	Yuhan Corporation
QL‐1005	GDF15/GLP1/GLP1R	Intermediate hyperglycaemia Obesity	Preclinical	NA	Beijing QL Biopharm Co Ltd
CIN‐209	GDF15/GLP1/GLP1R	Obesity	NA	NA	Janssen Pharmaceuticals Inc
FM9‐6 + Fc_hole	GDF15	Obesity	NA	NA	YuHan
IgG1‐GDF (CRL)	GDF15	Obesity	NA	NA	LG Chem Ltd
IgG4‐GDF (CRL)	GDF15	Obesity	NA	NA	LG Chem Ltd
FcA10 (−, L234A/L235A)‐(G4Q)4‐GDF15(N3Q/D5E)	GDF15	Obesity	NA	NA	Amgen
NGM‐386 (NP‐201)	GFRAL	Obesity	NA	NA	NGM Biopharmaceuticals Inc
GFANT‐01	GFRAL	Anorexia nervosa Obesity Cachexia Male sexual dysfunction nausea and vomiting	NA	NA	Syracuse University
GFANT‐05	GFRAL	Anorexia nervosa Obesity Cachexia Male sexual dysfunction nausea and vomiting	NA	NA	Syracuse University

Abbreviation: NA, not available. Data acquired from https://pharma.bcpmdata.com/ and https://clinicaltrials.gov/.

### 11.8 QL1005

QL1005, a dual agonist for GDF15 and GLP1, is generated by fusing GLP1 variant to the N terminus of the GDF15 analogue through a specific peptide linker, and the drug is also chemically conjugated C‐18 fatty di‐acid for time–action protraction. In cellular experiments, QL1005 effectively triggered the activation of both GLP1 and GDF15 receptors. In trials conducted with obese mice, QL1005 treatment resulted in decreases in body weight, food intake, insulin levels, fasting glucose and triglycerides. Similarly, in the crab‐eating monkey model of obesity, QL1005 exhibited dose‐dependent reductions in body weight, food intake, insulin and glucose, all while exhibiting minimal occurrences of gastrointestinal side effects. Notably, QL1005 demonstrated superior pharmacodynamic effects compared to single‐target medications such as samaglutide or long‐acting GDF15. As a result, QL1005 is expected to emerge as a promising contender for addressing obesity and its related metabolic disorders [104].

### 11.9 CIN‐209

CIN‐209, another dual GDF15/GLP1 agonist, was developed by Janssen Pharmaceuticals Inc. However, little is known about this drug publicly (Table 1). There is great anticipation for the results of future clinical trials about these drugs.

## 12 Side Effects of Exogenous GDF15

GDF15 has shown promising effects as a potential therapeutic target in animal studies and preclinical research, particularly in controlling appetite and body weight. However, the use of exogenous GDF15 is also associated with some side effects, primarily related to its mechanisms of appetite regulation and energy metabolism. The following are potential side effects that may arise from the usage of exogenous GDF15.

### 12.1 Nausea and Vomiting

One of the main side effects of GDF15 is the induction of nausea and vomiting. This occurs because GDF15 activates neurons located in the brainstem via its receptor GFRAL. These brain regions are involved not only in appetite control but also in regulating nausea and vomiting. In animal experiments and preclinical studies, the administration of exogenous GDF15 is often accompanied by decreased appetite, along with responses of nausea and vomiting [71] (NCT05199090 and NCT04199351).

### 12.2 Gastrointestinal Discomfort

In addition to nausea and vomiting, GDF15 may cause various gastrointestinal symptoms, such as dyspepsia, diarrhoea or constipation (NCT05199090, NCT04199351). However, the specific mechanisms behind these symptoms are not yet fully understood.

### 12.3 Reduced Physical Activity

While GDF15 reduces food intake, it has also been found to suppress voluntary movement. This decrease in physical activity may negatively impact overall health, especially in patients who need to maintain a certain level of physical activity [60, 87].

### 12.4 Muscle Atrophy

Elevated levels of GDF15 in circulation are closely associated with muscle atrophy. In an in vitro experiment, treating C2C12 myotubes with GDF15 for 4 days resulted in a significant increase in the mRNA expression of muscle ring finger‐1 and atrogin, two genes related to muscle degradation, while cytosine rich protein 61 levels remained unchanged [112]. Additionally, overexpressing GDF15 in muscle led to a significant reduction in muscle mass compared to control‐treated muscles, suggesting that GDF15 may promote muscle atrophy [95]. However, the physiological role of the GDF15‐GFRAL pathway is not yet fully understood, and further research is needed.

In studies with hNAG‐1 transgenic mice, regardless of whether they were on an HFD, serum insulin‐like growth factor 1 levels consistently decreased, supporting the likelihood of skeletal muscle atrophy [75].

Although GDF15 holds great potential in the treatment of obesity and metabolic diseases, its side effects, particularly nausea, vomiting and gastrointestinal discomfort, remain major challenges in its clinical application. Future research needs to focus on optimising the pharmacological effects of GDF15 while minimising these adverse reactions to enhance its safety and acceptability in clinical treatment.

## 13 Conclusions and Future Perspectives

Indeed, recent studies have demonstrated that the GDF15 signalling pathway plays a vital role in the pathogenesis of obesity. Preclinical and clinical investigations utilising newly developed GDF15 protein analogues or the GFRAL agonists have shown promising antiobesity potential [103, 104]. However, there are still several important questions that need to be answered (Figure 2). Firstly, apart from mature GDF15 dimers, the amount and role of other forms of GDF15 in plasma and tissues are uncertain and it is unclear how these various forms may contribute to the overall effects of obesity. There is speculation that the GDF15 precursor may potentially have peripheral effects and exhibit anti‐inflammatory activity, but strong evidence is lacking. Additionally, the existence and characterisation of peripheral receptors for the GDF15 precursors are still unclear and need to be further explored. Another intriguing hypothesis is the potential beneficial effect of breaking down excess mature GDF15 dimers in plasma into GDF15 monomers. This might potentially transform waste into a valuable therapeutic substance for patients with accumulated mature GDF15. Given the potential of the GDF15 signalling pathway to modulate various critical metabolic pathways, it becomes imperative to elucidate novel mechanisms governing the interplay between GDF15 signalling and other metabolism‐related pathways in the context of obesity. This endeavour will deepen our understanding of the role of GDF15 in obesity and provide new directions for developing treatment strategies for obesity.

Obese children have been found to have lower levels of GDF15 (mean 79.5 ± 6.0 pg/mL) compared to normal‐weight children (mean 99.3 ± 10.8 pg/mL) [113]. However, in adults, the plasma GDF15 levels are higher in obese patients than in the normal population [59]. Is the function and mechanism of GDF15 consistent between obese children and adults? Does the preclinical experiment of weight loss drugs need to adjust the age of animals? Are weight loss drugs universal for adults or obese children? In the obese state, the expression of GDF15 in rodent and human adipose tissue appears to be inconsistent. There is also a lack of comparative studies between exogenous GDF15 and endogenous GDF15, so further research is necessary to fully elucidate the exact mechanisms and functional implications of altered GDF15 levels in obesity among different age groups.

Recent clinical trials targeting the GDF15‐GFRAL signalling have achieved moderate efficacy in obesity treatment [103, 107]. However, none have achieved the desired therapeutic outcomes. Given the differences in GFRAL receptor distribution between humans and animals, preventing interference from peripheral receptors during clinical trials could represent a new therapeutic approach. In the current research, there is little exploration into the binding sites of GDF15 and its receptors and further investigation of the binding mechanism seems to facilitate the development of multitarget drug combination therapy or multitarget drugs. Given that exercise can increase circulating GDF15 concentrations and thereby improve β‐cell function, whether a combination of endogenous and exogenous GDF15 would achieve more effective and healthier weight loss requires further study. Recently, the approval of GLP1 agonists by the FDA was a significant breakthrough in the treatment of obesity. Considering the independent antiobesity mechanism of GDF15 and GLP1, the development of multitarget drugs or combined drugs to reduce the dose seems to be a potential strategy to reduce the side effects of GLP1 agonists. Considering that obesity is a chronic and refractory disease, the patients should be offered a comprehensive approach that combines lifestyle therapy, pharmacotherapy and metabolic surgery.

## Author Contributions

**Jincheng Zhang:** software (lead), writing – original draft (lead), writing – review and editing (lead). **Jingquan Sun:** supervision (lead), writing – review and editing (lead). **Jielang Li:** writing – review and editing (equal). **Hongwei Xia:** conceptualization (lead), formal analysis (lead), funding acquisition (lead), methodology (lead), project administration (lead), resources (lead), supervision (lead), writing – original draft (lead), writing – review and editing (lead).

## Conflicts of Interest

The authors declare no conflicts of interest.

## Data Availability

No data were used for the research described in the article.
